# A Cross-Sectional Study of ‘Yaws’ in Districts of Ghana Which Have Previously Undertaken Azithromycin Mass Drug Administration for Trachoma Control

**DOI:** 10.1371/journal.pntd.0003496

**Published:** 2015-01-29

**Authors:** Rosanna Ghinai, Philip El-Duah, Kai-Hua Chi, Allan Pillay, Anthony W. Solomon, Robin L. Bailey, Nsiire Agana, David C. W. Mabey, Cheng-Yen Chen, Yaw Adu-Sarkodie, Michael Marks

**Affiliations:** 1 Clinical Research Department, London School of Hygiene and Tropical Medicine, London, United Kingdom; 2 Department of Clinical Microbiology, School of Medical Sciences, Kwame Nkrumah University of Science and Technology, Kumasi, Ghana; 3 Laboratory Reference and Research Branch, Division of STD Prevention, National Center for HIV/AIDS, Viral Hepatitis, STD and Tuberculosis Prevention Centers for Disease Control and Prevention, Atlanta, Georgia, United States of America; 4 Hospital for Tropical Diseases, University College London Hospitals NHS Trust, London, United Kingdom; 5 Public Health Division, Ghana Health Service, Accra, Ghana; Centers for Disease Control and Prevention, UNITED STATES

## Abstract

Yaws, caused by *Treponema pallidum* ssp. *pertenue*, is reportedly endemic in Ghana. Mass distribution of azithromycin is now the cornerstone of the WHO yaws eradication campaign. Mass distribution of azithromycin at a lower target dose was previously undertaken in two regions of Ghana for the control of trachoma. Ongoing reporting of yaws raises the possibility that resistance may have emerged in *T. pallidum* pertenue, or that alternative infections may be responsible for some of the reported cases. We conducted a cross-sectional survey in thirty communities in two districts of Ghana where MDA for trachoma had previously been conducted. Children aged 5–17 years with ulcerative lesions compatible with yaws were enrolled. Samples for treponemal serology and lesion PCR were collected from all children. 90 children with 98 lesions were enrolled. Syphilis serology was negative in all of them. PCR for *T. pallidum* ssp *pertenue* was negative in all children, but *Haemophilus ducreyi* DNA was detected in 9 lesions. In these communities, previously treated for trachoma, we found no evidence of ongoing transmission of yaws. *H. ducreyi* was associated with a proportion of skin lesions, but the majority of lesions remain unexplained. Integration of diagnostic testing into both pre and post-MDA surveillance systems is required to better inform yaws control programmes.

## Author Summary

Yaws is a chronic bacterial infection of the skin and bones that is endemic in Ghana. Since 2012, mass distribution of the antibiotic azithromycin has become the cornerstone of the WHO yaws eradication campaign. Accurate surveillance data are necessary to guide decisions about where MDA is required. Many national surveillance systems report only clinical cases without laboratory confirmation, which may lead to misdiagnosis. Mass distribution of azithromycin, at a lower dose than is used for yaws, is also used in the control of the eye disease trachoma. Between 2001 and 2008, two regions of Ghana undertook distribution of azithromycin to eliminate trachoma. These regions still report cases of yaws, and it is unclear if these are due to drug resistant yaws or other causes. We conducted a survey of patients with skin ulcers that looked like yaws in one of these regions of Ghana. Blood tests for yaws were negative in all the individuals examined and molecular testing of the ulcers did not show evidence of yaws caused by drug resistant *Treponema pallidum* ssp. *pertenue*. Our data suggest that previous treatment for trachoma may have also treated yaws in these districts. These findings highlight the need for yaws control programmes to integrate diagnostic testing into surveillance.

## Introduction

Yaws is a re-emerging endemic treponemal infection caused by *Treponema pallidum* ssp. *pertenue* [1]. Early disease is characterised by papillomatous and ulcerative skin lesions, whilst late stage disease is characterised by skin lesions, bony involvement, and rarely progression to destructive lesions of the nasopharynx. Azithromycin (30mg/kg, maximum dose 2g) has recently been shown to be effective in the treatment of yaws and is now central to WHO’s yaws eradication strategy [2, 3]. Azithromycin is also key to the “SAFE” strategy for trachoma elimination. The target dose used in trachoma mass treatment (20mg/kg, maximum dose 1g) is lower than that demonstrated to be effective against yaws [4] although the delivered dose is frequently higher when a height based dosing strategy is used [5]. Although resistance has yet to be documented in yaws, azithromycin resistance has emerged in other treponemal infections, notably syphilis [6–9]. The development of drug resistant *T. pallidum* ssp *pertenue* would have significant implications for yaws eradication ambitions.

**Figure 1:**
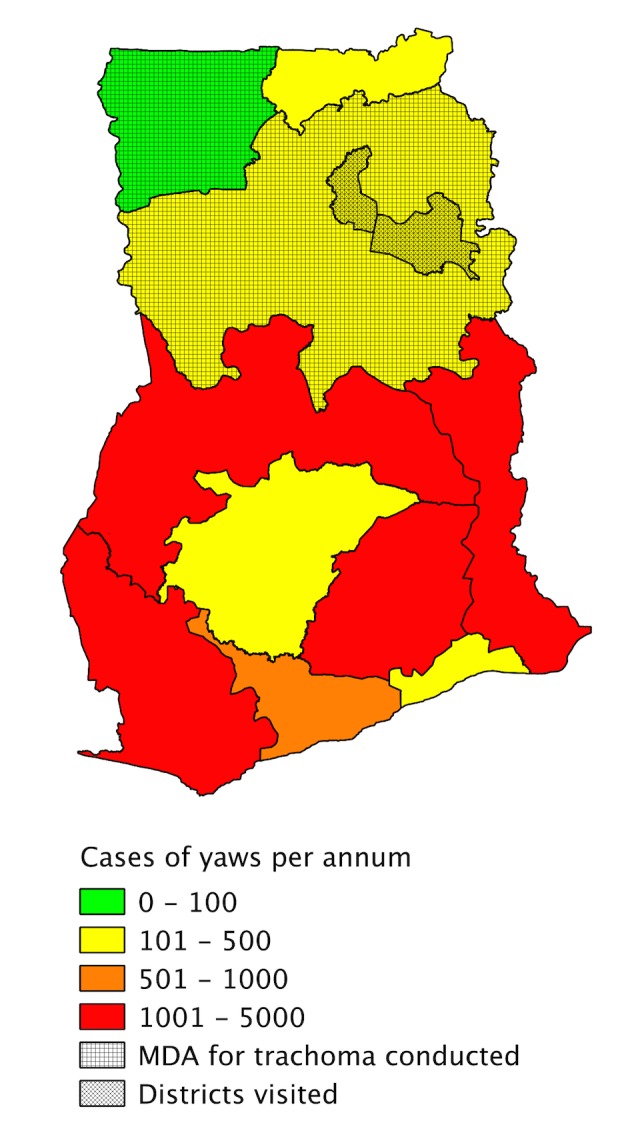
Map of Ghana showing number of yaws cases reported by the national surveillance system in 2012 and the regions where azithromycin mass drug administration for trachoma was conducted between 2001 and 2008.

In the 1950s–60s and 1980s, two large yaws control programmes using penicillin were conducted in Ghana, successfully reducing the burden of the disease, but yaws has subsequently re-emerged throughout the country and Ghana remains a major focus of transmission in Africa. Over 20,000 clinical cases were reported there in 2010 [10]. Ghana undertook azithromycin mass drug administration for the control of trachoma between 2001 and 2008 in the Northern and Upper West regions of the country [11]. Both regions continue to report cases of yaws through the national surveillance system (Fig. 1).

The epidemiology of yaws has been further complicated by the discovery that *Haemophilus ducreyi*, the causative agent of the ulcerative sexually transmitted disease chancroid, is a common cause of non-genital skin lesions in several countries where yaws is endemic [12, 13]. This makes the accuracy of surveillance data based on clinical case detection alone, without serological or molecular diagnostics, uncertain. A wide range of other organisms may cause ulcerative skin lesions, including *Mycobacterium ulcerans*, the causative agent of Buruli Ulcer, which is endemic in Ghana.

There is an urgent need to obtain accurate data on the epidemiology of yaws to guide disease eradication efforts. Ongoing reporting of yaws in Ghana, despite several rounds of azithromycin MDA, raises the possibility that resistance may have emerged in *T. pallidum* ssp. *pertenue* or that alternative causes may be responsible for some or all of the cases reported in some regions of the country. As current information suggests that Ghana is a major focus of yaws worldwide, either finding would have implications for the WHO yaws eradication campaign.

We conducted a yaws survey in two districts of Ghana in which MDA for trachoma was previously undertaken, and from which cases of yaws are still reported via the national surveillance system. We hypothesised that both *H. ducreyi and* azithromycin-resistant strains of *T. pallidum* ssp *pertenue* would be responsible for clinical cases of yaws seen in these districts.

## Methods

### Survey Design

We conducted a cross-sectional observational survey of moist ulcers suspicious for yaws in two post-azithromycin-MDA districts of northern Ghana, in the rainy season of June–July 2014. Yendi and Savelugu-Nanton undertook four and six annual rounds of azithromycin MDA for elimination of blinding trachoma, respectively, between 2001 and 2007 and both still report cases of yaws to the Regional Health Directorate.

### Patient Recruitment

In each community visited, children attending school (aged 5 to 17 years) were screened for the presence of moist ulcers suspicious for yaws according to the WHO yaws pictorial guide [14]. Children with other features of yaws were not enrolled, unless they also had a moist or exudative ulcer from which a lesion swab could be obtained.

### Study Procedures

For each participant we conducted a standardised interview including information about duration and location of skin lesions and presence or absence of bone symptoms compatible with secondary yaws. Children then underwent a standardised examination including examination of all skin lesions and for evidence of secondary yaws. For each ulcer, an impression of the shape of each moist ulcer was recorded, followed by measurement of its longest and shortest axes. Ulcers were classified as acute if they had been present 28 days or less, and as chronic if they had been present for longer. Ulcers were classified as deep if there was subcutaneous involvement; and tender if palpation prompted an involuntary expression of pain. Slough or granulation in the ulcer base and induration of ulcer edges were also recorded [13]. We systematically examined patients for the presence of regional lymphadenopathy.

A serum sample was collected from each patient for syphilis serological testing. Each exudative ulcer was swabbed with a single sterile swab. The swab was then placed onto an FTA Elute Micro Card (GE Healthcare, Buckinghamshire, UK) using three side-to-side motions, whilst pressing down firmly. Cards were placed inside separate re-sealable plastic packets with a sachet of desiccant. The ulcer was dressed to deter flies. Following completion of study procedures each participant was weighed, and the appropriate single oral dose of azithromycin offered for empirical treatment of yaws.

### Laboratory Testing

Serum samples were stored at –20°C and transferred to Komfo Anokye Teaching Hospital (KATH), Kumasi, where they were thawed and tested using the *Treponema pallidum* particle agglutination (TPPA)(Mast Diagnostics, Merseyside, UK) and qualitative rapid plasma reagin (RPR) tests (Deben Diagnostics, Ipswich, UK). Samples that were positive on qualitative RPR testing were titrated out to obtain a quantitative RPR. Serology was considered positive for yaws if the TPPA was positive and the RPR titre was ≥1:8. All serological tests were conducted in duplicate. 10% of serum samples, selected at random, were retested by an independent laboratory scientist for quality control purposes.

All swab samples were transferred to the Centers for Disease Control and Prevention (CDC) where DNA was extracted and subjected to real-time PCR for the detection of *T. palladium* ssp. *pertenue*, as previously described [15]. If any samples tested positive for *T. pallidum* a second real-time PCR for the A2058G and A2059G mutations associated with macrolide resistance [16] was ready to be applied. Regardless of the result of *T. pallidum* ssp. *pertenue* testing, all samples were additionally tested with a second multiplex PCR for *H. ducreyi* and *M. ulcerans* DNA, using previously validated targets [17, 18].

### Statistical Analysis

We compared the clinical features and serological results of the children with yaws, *H. ducreyi*, and dual infections using Fisher’s exact test. All analyses were performed using Stata 13.1 (Statacorp, Texas).

### Ethics

Informed written consent was obtained from each child’s parent or guardian, and assent was gained from children by staff fluent in local dialects. The research protocol was approved by the London School of Hygiene & Tropical Medicine (LSHTM) (ref. no. 8202), the Kwame Nkrumah University of Science and Technology (KNUST) Committee on Human Research, Publication and Ethics (ref. CHRPE/AP/225/14), and the CDC’s National Center for HIV/AIDS, viral hepatitis, STD and TB prevention (NCHHSTP) Associate Director for Laboratory Science (ref. no. 6634).

## Results

Approximately 3,000 children from thirty communities were screened for inclusion in the study. 92 children (with a total of 100 moist ulcers) from a total of thirty communities were enrolled in the study. Two children (with one ulcer each) were later found to be ineligible (the first was over 18 years, and the second was from a community that did not undergo azithromycin MDA for trachoma), and were excluded from the results, leaving 90 children with a total of 98 moist ulcers. Many children had evidence of other skin problems but there was no evidence of typical papillomatous, palmar-plantar or macular lesions of primary or secondary yaws. The median age of enrolled children was 10 years (IQR 8 to 12 years), and 68 subjects (76%) were male. Thirty-seven children (41%) had not been born or were aged less than 1 at the time MDA was completed in their district (Table 1).

**Table 1:** Baseline characteristics of study participants.

	**Overall n = 90**	**Yendi District n = 46 (51%)**	**Savlugu-Nanton District n = 44 (49%)**
Male sex	68 (76%)	33 (72%)	35 (83%)
Age (years)			
5–8	28 (30.4%)	12 (26%)	16 (37%)
9–12	50 (55.6%)	27 (59%)	23 (52%)
13–17	12 (14%)	7 (15%)	5 (11%)
Eligible for MDA*			
No rounds of MDA*	37 (41%)	9 (20%)	28 (64%)
At least 1 round of MDA*	53 (59%)	37 (80%)	16 (36%)
All rounds of MDA*	19 (21%)	16 (35%)	3 (7%)

* MDA was conducted in Yendi District between 2004 and 2007. MDA was conducted in Savelugu-Nanton district between 2001–2004. Individuals were considered eligible for at least 1 round of MDA if they were aged 1 or over the final year MDA was conducted in their district. Individuals were considered eligible for all rounds of MDA if they were aged 1 or over in the first year MDA was conducted in their district.

**Figure 2:**
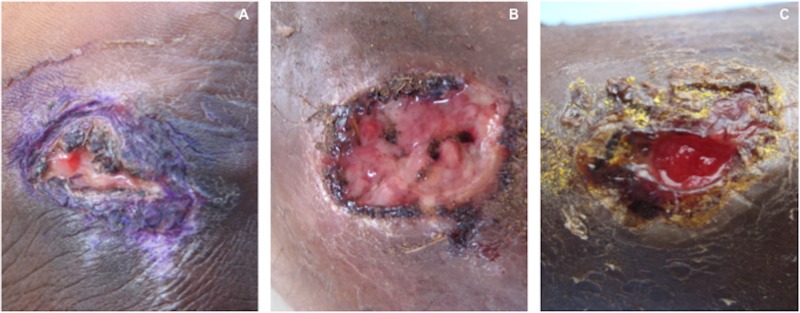
Ulcerative lesions to which with local treatment have been applied. a) Lesion treated with gentian violet paint. b) Lesion treated with animal excrement. c) Lesion treated with ‘topaya’—the content of an antibiotic capsule.

The majority of ulcers sampled, 91/98 (93%), were on the lower legs, and 54/98 (55%) were painful. There was a balance of acute (53/98, 54%) and chronic lesions (45/98, 46%). Many participants reported applying local topical treatments to skin lesions, including gentian violet paint, animal faeces or the contents of antibiotic capsules known as” topaya” or “bullets” (Fig. 2). Bone or joint symptoms and signs were noted in 54/92 individuals (59%). Although mild lymphadenopathy was common, no participant had clinically significant regional lymphadenopathy.

Serology for treponemal infections (both TPPA and qualitative RPR) was non-reactive in all individuals. Quantitative RPR was not performed, as no samples were positive with qualitative RPR. Using real-time PCR, *T. p. pertenue* DNA was not detectable in any lesion sample. *H. ducreyi* DNA was detected in 9/98 (9.2%) ulcers (from eight children). Two children infected with *H. ducreyi* had two moist ulcers: in one case *H. ducreyi* DNA was detected in only one ulcer, and in the other case, both ulcers were positive for *H. ducreyi* DNA. PCR was negative for all three pathogens in swabs from the majority (89/98, 91%) of ulcers. There was no significant difference in the clinical characteristics of *H. ducreyi* positive and *H. ducreyi* negative lesions (Table 2).

**Table 2:** Clinical Features of Ulcerative Lesions.

	**Lesions from which *Haemophilus ducreyi* DNA detected (n = 9)**	**Lesions from which *Haemophilus ducreyi* DNA not detected (n = 89)**	**Odds Ratio**	**p**
Male sex	7 (78%)	69 (78%)	1.01 (0.19–5.32)	0.9864
Age (median (inter-quartile range))	10 (8–11)	10 (8–12)	N/A	0.7466
Duration (mean weeks (inter-quartile range))	4.3 (2–12.9)	3 (1.4–4.3)	N/A	0.4831
Deep Ulcer	5 (56%)	45 (51%)	1.22 (0.31–4.89)	0.7763
Round Shape	3 (33%)	16 (18%)	2.28 (0.51–10.27)	0.2692
Tender Ulcer	9 (100%)	85(96%)	N/A	0.5182
Indurated Edges	5 (56%)	42 (47%)	1.40 (0.35–5.60)	0.6339
Lesion site in lower limb	9 (100%)	87 (98%)	N/A	0.6512

## Discussion

In this study conducted in the Northern Region of Ghana we found no evidence of active or latent yaws. All patients in this study had negative treponemal and non-treponemal serology. The fact that treponemal-specific serology was negative indicates that these individuals have never been infected with yaws. Both of the districts selected for this study had completed several rounds of azithromycin MDA for trachoma in the preceding decade. It is plausible that MDA for trachoma interrupted transmission of yaws resulting in its elimination in these districts and that these children, of whom about forty-percent never received MDA, have therefore never been exposed to yaws.

The original intention of this study had been to identify azithromycin resistant strains of *T. pallidum* ssp *pertenue* that may have emerged following MDA, given that a lower target dose of azithromycin is used in trachoma control programmes than is now recommended in yaws control programmes. The absence of any drug resistant strains of *T. pallidum pertenue* in an area with repeated exposure to lower dose azithromycin is encouraging for other settings where trachoma and yaws are co-endemic and may reflect the higher target dose of azithromycin that is normally achieved with a height based dosing strategy. An alternative possibility is that these regions were never endemic for yaws. Exploring this hypothesis would require large numbers of historic specimens and was beyond the scope of the current study.

Our study highlights the poor specificity of clinical case reporting and the urgent need to integrate serology into clinical surveillance systems. Yaws cases continue to be reported from both the districts we visited but our results show that these cases are extremely unlikely to represent yaws. A rapid diagnostic test for yaws has shown value both in community surveillance [19] and as a confirmatory test in clinically suspected cases [20] and our data strengthen the case for this test to be made available to national yaws programmes as soon as possible.

Results of PCR testing add further to the growing evidence that *H. ducreyi* is a cause of non-genital ulcerative skin lesions in children. This study is the first to report on the impact of testing multiple skin lesions from the same individual. Of those with multiple tested lesions, we identified some whose PCR results were concordant between lesions and others where PCR results were discordant between lesions: only testing one lesion may result in the false assumption that all of a patient’s lesions have the same aetiology. Whilst azithromycin has been shown to be effective at treating non-genital *H. ducreyi* in experimental models [21], it will be important to develop tools to monitor for the emergence of drug resistance in real-world settings.

Of equal significance is the large proportion of lesions for which no aetiological agent was identified. It is likely that many lesions were non-infectious, for example due to trauma or fly bites. Common bacterial infections such as *Staphylococcus aureus* may also have been responsible. Further study, including longitudinal data, will be needed to assess this question in more detail. Understanding the aetiology and epidemiology of non-yaws skin lesions is important for maintaining community engagement with mass drug administration programmes for the eradication of yaws.

Our study has a number of limitations. Most notably we only enrolled individuals with ulcerative lesions. It is possible that if we had also enrolled children with other skin lesions compatible with yaws, we may have identified some seropositive subjects. The negative TPPA testing in all individuals in our study however, suggests there is minimal transmission of treponemal diseases in children in this community. Secondly, we only tested skin lesions for a small number of aetiological agents, which limits our ability to fully characterise the cause of skin lesions in this community. Finally, whilst we know which districts were treated during the trachoma control programme and that an overall programmatic coverage of 93.9% was reported [22], there are limited data on the actual coverage achieved in the specific communities we visited in this study.

In summary, we found no evidence of ongoing transmission of yaws in two districts of Ghana, which had previously received MDA for trachoma. As in other countries, it is clear that *H. ducreyi* is responsible for clinically similar skin lesions but the majority of lesions remain unexplained. There is an urgent need to integrate serological testing into surveillance to better inform national reporting data and yaws control programmes.

## Supporting Information

S1 Checklist STROBE checklist. (DOCX)

S1 Dataset Study dataset. (CSV)
